# Co-pathogens in Periodontitis and Inflammatory Bowel Disease

**DOI:** 10.3389/fmed.2021.723719

**Published:** 2021-09-20

**Authors:** Zhengwen Cai, Tao Zhu, Fengshuo Liu, Zixuan Zhuang, Lei Zhao

**Affiliations:** ^1^State Key Laboratory of Oral Diseases, West China College of Stomatology, Sichuan University, Chengdu, China; ^2^Department of Gastrointestinal Surgery, West China Hospital, Sichuan University, Chengdu, China; ^3^Department of Periodontics, West China Hospital of Stomatology, Sichuan University, Chengdu, China

**Keywords:** periodontitis, inflammatory bowel disease, periodontal disease, bacteria, microbiota, intestine

## Abstract

Localized inflammatory lesions in one area of the body may affect other distant organs through various modes of transmission thus initiating secondary inflammatory infections. Periodontal disease (PD) and inflammatory bowel disease (IBD) have been shown to coexist. Periodontitis is a multifactorial inflammatory disease, and dental plaque is considered to be the initial risk factor. Individuals with genetic susceptibility are more likely to develop periodontitis when exposed to external stimuli. IBD is affected by host genetics, immunoregulation, daily diet, and the gut microbiota, and its risk factors appear to be shared with those of PD. However, the key etiologies of both diseases remain unclear, thus hindering the exploration of possible links between IBD and PD. Recent studies and systematic reviews have focused on evidence-based statistics of the prevalence and clinical manifestations of both diseases, but discussions of the microbial etiological correlation between periodontitis and intestinal inflammation are scarce. Here, we summarize the potential common pathogenic microorganisms that may serve as bridges between the two diseases. Studies have shown that invasive microorganisms such as *Porphyromonas gingivalis, Fusobacterium nucleatum, Klebsiella* spp. and *Campylobacter* spp. play key roles in the comorbidity of PD and IBD.

## Introduction

### What Is Periodontal Disease?

PD is an inflammatory disease that affects the periodontium and alveolar bone. Changes in the gingiva such as swelling and redness are often the earliest signs (1). If untreated, gingivitis can progressively deteriorate, leading to attachment loss, formation of periodontal pockets and alveolar bone loss, which are known as periodontitis (2). Severe periodontitis can result in loose or missing teeth and is reported to be a major cause of tooth loss in adults (3). However, the pathogenesis of periodontal disease remains uncertain. Both dental plaque and the balance between host immune responses and the microbiota are important initiating factors (4). PD is also associated with various systemic diseases, including diabetes mellitus (5), atherosclerosis (6), and adverse pregnancy outcomes (7), which affect each other bidirectionally. However, the relationship between periodontitis and inflammatory bowel disease (IBD) is unclear, and the precise mechanisms must be clarified.

### What Is IBD?

IBD is characterized by chronic recurrent intestinal inflammation and consists mainly of ulcerative colitis (UC) and Crohn's disease (CD). IBD morbidity has increased dramatically from the twentieth century (8), and its pathogenesis remains unclear. Studies suggest that environmental factors, genetic susceptibility, the intestinal microbiota, and immune responses are involved in IBD development (9, 10). Among these factors, intestinal microorganisms play key roles in IBD occurrence and progression (11). Varied metabolites are also involved in the changes in the intestinal ecosystem (12).

### Evidence-Based Association Between IBD and PD

Meta-analyses from evidence-based medicine have integrated several observational studies demonstrating that patients with periodontitis or IBD had an increased risk of also having the other disease, with a pooled odds ratio of 3–5 (13–15). Furthermore, a few cohort studies indicated that patients with IBD tended to have a higher risk of developing PD than did those without IBD (16). Patients with periodontitis also had greater risks of developing UC than did the controls (17, 18). Retrospective and cohort studies have confirmed a bidirectional association between IBD and PD. However, questions remain regarding how the two diseases interact with each other and what are their microbial causes and common risk factors.

### Roles of Microorganisms in PD and IBD

Studies have found no specific microbial pathogens for either periodontitis or IBD. Epidemiological statistics, clinical symptoms, and risk factors imply that some correlations, such as the common suspicious microorganisms, exist between periodontitis and IBD (15–17, 19–22). In the oral cavity, microbes gather and propagate at subgingival gaps, forming dental plaques (23, 24), which act as a pathogenic arsenal that can produce antigens to invade the gingival mucosa in the deep periodontal pockets. Furthermore, these pathogens can interfere with host immune defenses to exacerbate inflammation. For example, the red complex (*Porphyromonas gingivalis, Treponema denticola* and *Tannerella forsythus*) has been strongly associated with periodontitis (25). Research has demonstrated a correlation between *Porphyromonas gingivalis* infections (26) and a deficiency of Toll-like receptors, which decreases the strength of the innate immune system (27) and leads to an imbalance between invasive pathogens and host defenses in the periodontal tissues. This disrupts the homeostasis, leading to inflammation in the oral cavity.

Humans share many common gut microbes, such as Firmicutes and Bacteroidetes (28, 29). From infancy to adulthood, the resident intestinal microorganisms evolve and ultimately attain relative stability, signifying the maturity of the host's intestinal ecological system (28). The dynamic and relatively constant microbiota contributes to building intestinal homeostasis and shapes a biological barrier in the alimentary tract. Conversely, dysbiosis in the gastrointestinal tract induces inflammation in susceptible hosts (30). Fecal microbiota transplantation alleviates UC symptoms in patients with UC, suggesting that alteration of the intestinal microbes affects the outcomes of IBD (31).

## Correlation Between Periodontitis and IBD

### Prevalence and Comorbidity

Several studies have focused on the increasing comorbidity of periodontitis and IBD (15–17). These clinical evidence-based studies can be divided into two categories: (1) patients with IBD who have a higher risk of developing periodontitis and (2) patients with periodontitis who have a higher risk of developing IBD. One cross-sectional study of 1,297 patients reported a 30% higher risk of periodontitis in patients with IBD than in controls without IBD (32). Another investigation showed that patients with IBD had deeper periodontal pockets, less clinical attachment, and more severe gingival bleeding compared with those of the healthy controls (33). Retrospective statistics from the National Health Insurance Research Database in Taiwan revealed that patients with periodontitis had a higher risk of IBD than did matched controls without periodontitis (17). Additionally, patients with periodontitis had a greater risk of subsequent UC than did the controls (17). In treatment, some pharmacotherapies for CD may protect patients against periodontitis, suggesting a relationship between IBD and PD (16).

### Microbiological Associations Between Periodontitis and IBD

**Figure 1:**
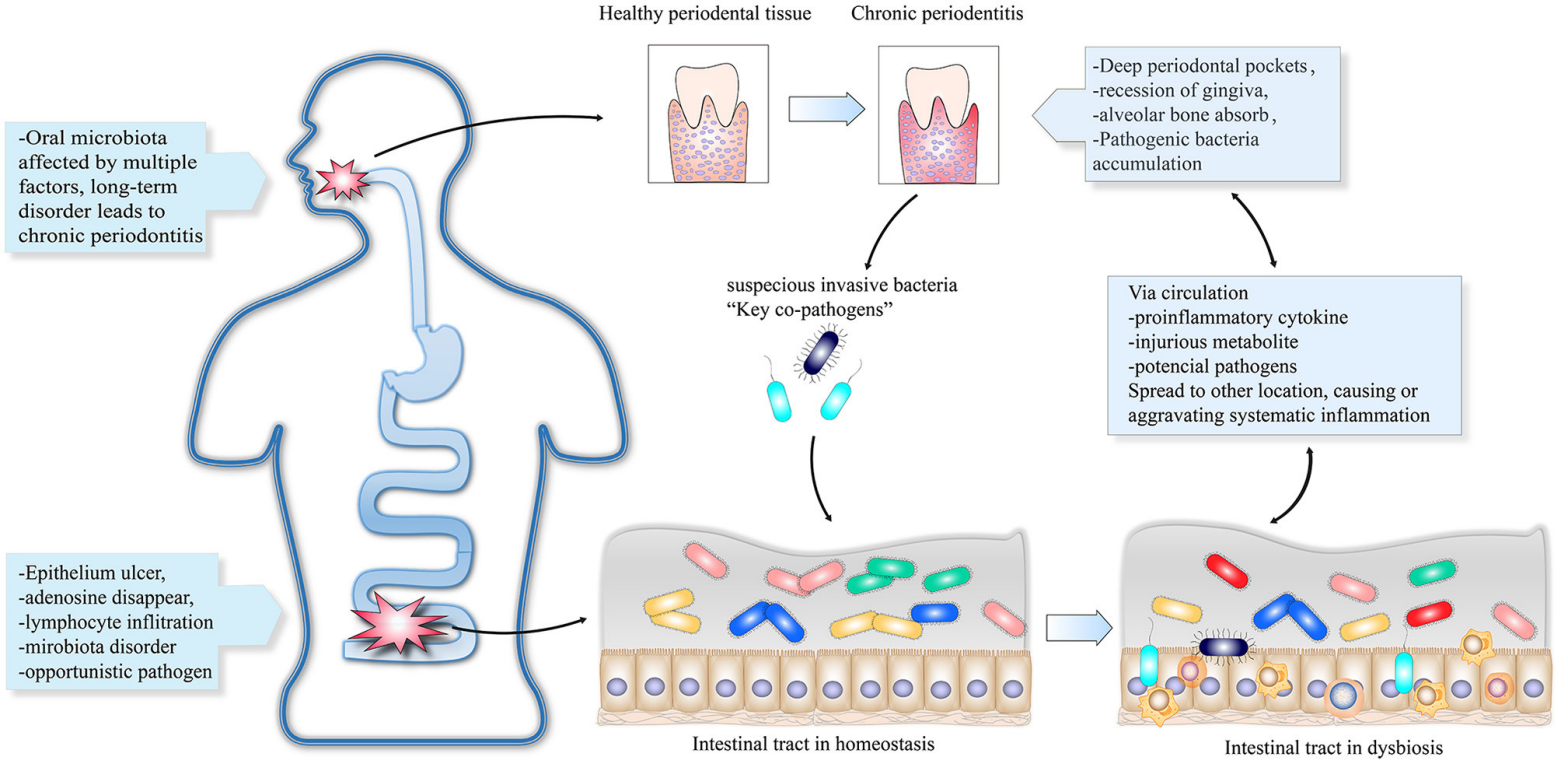
The oral cavity and intestinal tract can contain initial lesions infected with suspicious invasive bacteria that lead to localized dysbiosis. The key pathogens can travel through the digestive tract to other parts of the body and trigger or aggravate inflammatory diseases. Other proinflammatory cytokines and harmful metabolites can be transmitted *via* the circulatory system to initiate inflammation.

Compositions of normal bacteria, opportunistic pathogens, pathogenic microorganisms, and probiotics affect the microecological balance. An imbalanced microecology can further lead to disease (34). Oral microbes can be transmitted to the gut *via* the gastrointestinal tract (35). This provides the basis for translocation of pathogenic bacteria from the oral cavity to the gut, thus disrupting the intestinal homeostasis. One hypothesis suggests that suspicious pathogens promote the co-occurrence of PD and IBD. First, periodontal pathobionts migrate from the oral cavity to the gut and lead to dysbiosis of the intestinal ecology. Second, the disordered state of the gut microbiota triggers an intestinal immune response, which manifests as intestinal and systemic inflammation leading to the occurrence or aggravation of periodontitis. Finally, dysbiosis of either the oral or intestinal microbiota can be initial causes of PD or IBD. Subsequent alterations, including microorganisms, virulence factors, harmful metabolites, and other proinflammatory factors, can spread between both the intestines and the oral cavity *via* the circulatory system. Figure 1 shows the bidirectional reinforcement cycle in the deterioration of PD and IBD. However, no specific pathogenic strain has been identified. We propose several PD- and IBD-associated microorganisms that may be key causative agents for a higher risk of IBD and PD comorbidity.

#### Porphyromonas Gingivalis

*Porphyromonas gingivalis* is considered a critical pathogen in periodontitis (19) as well as a risk factor for enteric inflammation *via* altering the microbiota composition and metabolite profiles (20). Mice who were orally administered *P. gingivalis* exhibited increased levels of amino acid metabolism, including biosynthesis of phenylalanine, glutamine, tyrosine, and tryptophan in the gut and serum, implying that oral administration of *P. gingivalis* induced alterations of the gut microbiota composition and metabolites (20). Jia et al. (36) found that *P. gingivalis* upregulated the Th17-associated transcription factor, RoRγt, and increased the IL-17 and IL-6 levels. Conversely, *P. gingivalis* downregulates the expression of Treg transcription factor Foxp3, TGF-β, and IL-10 *via* the TLR4 pathway. These authors revealed the relationship between *P. gingivalis* and IBD through a dextran sodium sulfate (DSS)-induced IBD mouse model in which *P. gingivalis* activated CD4+ T cells and exacerbated colitis by upregulating the Th17/Treg ratio *via* the JAK-STAT signaling pathway (36). Tsuzuno et al. (37) found a significant exacerbation of colitis in DSS-induced mice administered *P. gingivalis*. These authors reported that *P. gingivalis* reduced the tight junction proteins by decreasing zonula occludens-1 (ZO-1) levels in intestinal epithelial cells, which in turn disrupted the intestinal barrier function. Additionally, They found that *Prevotella intermedia* (38) and *Fusobacterium nucleatum* (39), which are considered periodontopathic bacteria, also exacerbated colitis (37). Although the exacerbation was to a lesser extent than that of *P. gingivalis*, and the exacerbation mechanism did not occur *via* disruption of the tight junctions of the intestinal epithelial barrier, *P. intermedia* and *F. nucleatum* may act as links between periodontitis and IBD and have a synergistic effect in intestinal inflammation (37).

#### *Klebsiella* spp.

*Klebsiella* spp. are oral pathogens that ectopically colonize the gut and induce dysbiosis and inflammation (21, 22). Atarashi et al. (21) gavaged gnotobiotic mice with saliva from patients with CD and analyzed the microbial differences between the saliva and mouse feces *via* 16S rRNA sequencing. It was found that *Klebsiella pneumoniae 2H7* can ectopically colonize the intestines *via* the oral cavity and significantly induce Th1 cell responses, suggesting that some oral pathogenic bacteria can exacerbate intestinal diseases. Kitamoto et al. (40) found that *Klebsiella* and *Enterobacter* spp. triggered colitis in mice with ligature-induced periodontitis (41). Periodontitis can trigger and exacerbate intestinal inflammation *via* the direct microbial pathway and the indirect immunological pathway. To verify the direct pathway, Kitamoto et al. used mouse models of ligature-induced periodontitis and DSS-induced colitis and found that the colitis was more severe in the ligature+DSS model than in the DSS-alone group. High-throughput sequencing technology was used to determine the significantly different *Klebsiella* and *Enterobacter* spp. (40). The bacteria were isolated, cultured, and tested in germ-free *Il10*^−/−^ mice and specific pathogen-free mice. The oral pathogenic *Klebsiella* and *Enterobacter* spp. were transferred and colonized the intestines through the digestive tract *via* the direct pathway, thus stimulating macrophages to secrete IL-1β *via* caspase-11-mediated inflammasomes to induce colitis (40). To confirm the indirect pathway, Kitamoto et al. used Kaede fluorescent protein mice to track T-cell migration (42) and found that orally primed T cells migrated to the colonic lamina propria only in the ligature + DSS-induced model mice (40). Neither ligature nor DSS alone resulted in the same phenotype. Thus, the reactive Th17 cells were activated immunologically by oral pathogens and migrated from the mouth to the intestines *via* the lymph nodes, where they were activated by homologous pathogens from the oral cavity, leading to intestinal inflammation (40).

#### *Fusobacterium* spp.

*Fusobacterium nucleatum* plays an important role in dental plaque and periodontitis formation (39). This microorganism also resides in the intestinal tract and is associated with IBD, especially in patients with UC (43, 44). Huh and Roh (45) analyzed longitudinal metagenomic data from the integrative Human Microbiome Project (iHMP) and revealed that *F. nucleatum* may be associated with early intestinal dysbiosis and could serve as a biomarker for detecting IBD. Strauss et al. (46) found that *Fusobacterium* spp. could be isolated from 63.6% of patients with gastrointestinal disease compared with 26.5% of healthy controls (*P* = 0.01). *Fusobacterium nucleatum* strains derived from inflamed biopsy tissue from patients with IBD were more invasive than those isolated from healthy tissue from patients with IBD or from controls (*P* < 0.05), revealing that *F. nucleatum* in the oral cavity may be a source of highly invasive bacteria in IBD (46). Liu et al. (47) reported that *F. nucleatum* can exacerbate IBD by damaging epithelial integrity and increasing permeability by regulating expression of the tight junction proteins zonula occludens-1 and occludin. It also promotes secretion of cytokines such as IL-1β, IL-6, and IL-17 and induces CD4(+) T-cell proliferation by activating the STAT3 signaling pathway.

#### *Campylobacter* spp.

*Campylobacter* spp. are present in the oral cavity and alimentary tract of IBD patients and are considered causative agents of oral and intestinal diseases (48, 49). Hsu et al. (50) sequenced the genome biology of *Campylobacter showae* and identified the functions of type IV secretion machinery and S-layer proteins in invasive strains. These authors compared the strains isolated from the gut with those from oral supragingival plaques and found that they shared the same specific genes, indicating a similar potential virulence and pathogenic pathway in initiating periodontitis and IBD (50). Furthermore, *Campylobacter concisus* genes isolated from the same patients' gut and oral environments were similar, indicating relocation of oral microbes to the intestinal tract and the role of periodontopathogens in gastrointestinal disease (51).

**Figure 2:**
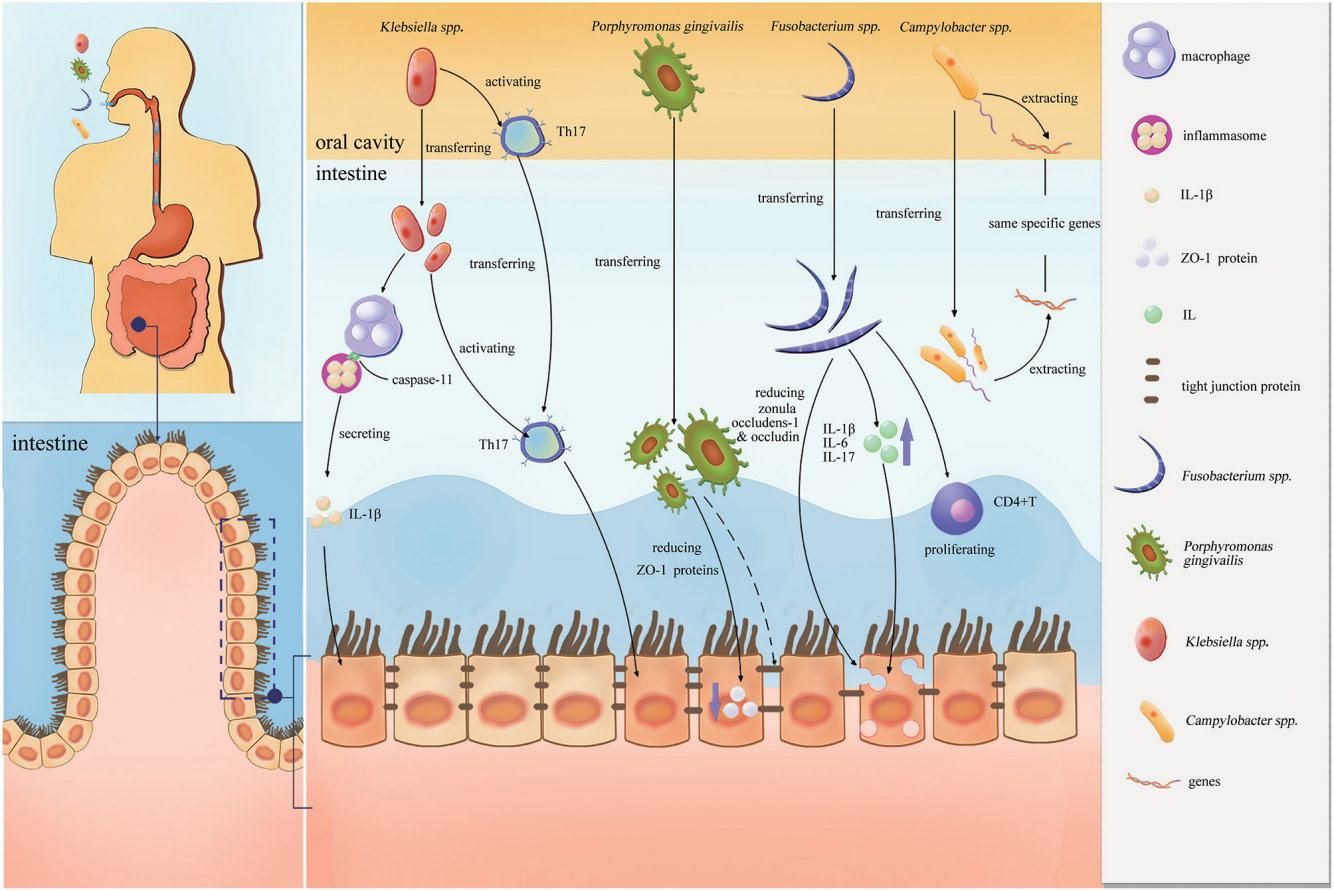
Proinflammatory effects of the four pathobionts (*Porphyromonas gingivalis, Klebsiella* spp., *Fusobacterium* spp., and *Campylobacter* spp.) in the intestines.

Figure 2 shows the proinflammatory effects of these four pathobionts (*Porphyromonas gingivalis, Klebsiella* spp., *Fusobacterium* spp., and *Campylobacter* spp.) in the intestines.

## Discussion

Periodontitis and IBD exhibit a high risk of co-occurrence and are potentially correlated. Studying diseases with interactions is challenging, and an inadequate understanding of IBD and PD further hinder this research. Dysbiosis of the microbiota is a common feature of both diseases. Dysbiosis of the oral microflora can trigger PD, while dysbiosis of the gut microflora contributes to IBD. Multiple risk factors, including dietary intake, smoking habits, oral hygiene, and host genetic susceptibility also influence oral and intestinal homeostasis (52, 53).

We emphasize the critical roles of suspicious pathobionts in both diseases; however, host immune factors are equally important. Susceptible individuals are more likely to experience dysbiosis and immune inflammatory responses triggered by pathogenic microorganisms. Immune cells and inflammatory cytokines can be transferred from areas of the body to other distant organs *via* the blood circulation and trigger subsequent diseases (39, 54). Cytokine expressions can be measured as observational indicators to evaluate the conditions of patients with periodontitis and IBD. Higher IL-1β, IL-4, IL-6, IL-10, and IL-21 expressions were detected in gingival tissues in patients with active IBD compared with those of patients in remission. Proinflammatory cytokine expressions are also positively correlated with disease severity scores (55). The IL-17/IL-23 axis appears to play critical roles in IBD and periodontitis by inducing and regulating the innate immune response to the tissues and pathogens (56, 57). A clinical control trial showed that periodontitis patients with concurrent IBD treated with anti-tumor necrosis factor alpha (anti-TNF-α) therapy had a higher probability of healing than did those managed without anti-TNF-α therapy (58). Transcriptomic analysis of periodontitis identified a special upregulated gene, pleckstrin, which was overexpressed in patients with UC and other chronic inflammatory diseases, supporting the hypothesis of a network between periodontitis and IBD (59).

Conversely, a cohort study by Yin et al. (60) reported an inverse relationship between poor oral health and IBD owing to the hygiene hypothesis (early poor oral hygiene can better induce immune tolerance in the gut, resulting in a lower IBD incidence). However, this may not be contradictory to the increased comorbidity of PD and IBD. Poor oral hygiene differs from periodontitis. Studies have shown that poor oral conditions can lead to a build-up of dental plaque, which in turn leads to gingivitis, but only a portion of gingivitis progresses to periodontitis (61, 62). Thus, poor oral hygiene is a risk factor for periodontitis rather than a direct cause. Yin et al. reported that poor oral hygiene in early childhood may induce immune tolerance, which leads to a lower probability of developing IBD in adulthood. This is distinct from our discussion of the increased risk of IBD in adults with poor oral hygiene and PD. If the hygiene hypothesis is true, individuals with good oral hygiene in early childhood (who do not develop an appropriate immune tolerance) may have a higher chance of developing IBD if they have poorer oral hygiene in adulthood.

## Conclusions

We emphasize the following four points:

1. Periodontitis and IBD have an increased probability of coexisting.
2. Multiple common features suggest a possible bidirectional relationship between periodontitis and IBD.
3. Certain invasive microorganisms (e.g., *Porphyromonas gingivalis, Fusobacterium nucleatum, Klebsiella* spp., and *Campylobacter* spp.) may play key roles in the comorbidity of PD and IBD.
4. Potential pathogenic microorganisms, immune responses, and other risk factors contribute to the link between PD and IBD, reinforcing the bidirectional cycle in the deterioration of the two diseases.

Increasing evidence implies a correlation between IBD and periodontitis. Patients with IBD can exhibit extraintestinal oral manifestations associated with PD, which can occur before intestinal inflammation and suggest the existence or risk of IBD (63). The severity and risk of developing periodontitis are higher for patients with IBD compared with those of people without IBD (15, 64). Likewise, patients with periodontitis have a higher prevalence of IBD (17, 18). Researchers should evaluate the oral cavity and the intestines simultaneously to more purposefully seek possible co-pathogens in the comorbidity of IBD and PD. This will provide a biomarker for diagnosing both IBD and PD. For patients with only the clinical manifestations of either IBD or PD, this biomarker may remind clinicians of the higher risk of comorbidity of both diseases for purposes of prevention. In the therapeutic field, it may provide target pathogens for the treatment of PD and IBD.

## Author Contributions

ZC and LZ contributed to conception and design. ZC, TZ, FL, and ZZ contributed to drafting and figure. ZC, TZ, FL, ZZ, and LZ contributed to manuscript revisions. All authors contributed to the article and approved the submitted version.

## Funding

This study was supported by a research grant from West China Hospital of Stomatology (LCYJ2019-4).

## Conflict of Interest

The authors declare that the research was conducted in the absence of any commercial or financial relationships that could be construed as a potential conflict of interest.

## Publisher's Note

All claims expressed in this article are solely those of the authors and do not necessarily represent those of their affiliated organizations, or those of the publisher, the editors and the reviewers. Any product that may be evaluated in this article, or claim that may be made by its manufacturer, is not guaranteed or endorsed by the publisher.
